# Intein-Mediated Protein Engineering for Cell-Based Biosensors

**DOI:** 10.3390/bios12050283

**Published:** 2022-04-28

**Authors:** Chungwon Kang, Keshab Lal Shrestha, San Kwon, Seungil Park, Jinsik Kim, Youngeun Kwon

**Affiliations:** Department of Biomedical Engineering, Dongguk University, Seoul 04260, Korea; iu8974@dgu.ac.kr (C.K.); ks029@dgu.ac.kr (K.L.S.); jqk567@dgu.ac.kr (S.K.); tmddlf0816@dongguk.edu (S.P.)

**Keywords:** biosensors, cell-based sensors, bio-recognition element, reporter element, split-intein, conditional protein splicing

## Abstract

Cell-based sensors provide a flexible platform for screening biologically active targets and for monitoring their interactions in live cells. Their applicability extends across a vast array of biological research and clinical applications. Particularly, cell-based sensors are becoming a potent tool in drug discovery and cell-signaling studies by allowing function-based screening of targets in biologically relevant environments and enabling the in vivo visualization of cellular signals in real-time with an outstanding spatiotemporal resolution. In this review, we aim to provide a clear view of current cell-based sensor technologies, their limitations, and how the recent improvements were using intein-mediated protein engineering. We first discuss the characteristics of cell-based sensors and present several representative examples with a focus on their design strategies, which differentiate cell-based sensors from in vitro analytical biosensors. We then describe the application of intein-mediated protein engineering technology for cell-based sensor fabrication. Finally, we explain the characteristics of intein-mediated reactions and present examples of how the intein-mediated reactions are used to improve existing methods and develop new approaches in sensor cell fabrication to address the limitations of current technologies.

## 1. Introduction

Biosensors are analytical devices that can screen for biological molecules of interest and monitor their interactions. Since the basic concept of the biosensor was first described by Leyland Clark in 1962, the field of biosensors has been growing at an accelerated rate [1]. Biosensors have now become a valuable tool not only for fundamental studies of complex biological problems but also for various applications including disease diagnostics, drug screening, and environmental monitoring. Conventional in vitro analytical biosensors are widely utilized, as they offer high selectivity and sensitivity with a fast response time [2,3]. However, despite its usefulness, in vitro sensor technology has important drawbacks. First, the biomolecular interaction of purified probes and targets may not properly represent the interactions taking place in vivo [2]. Additionally, in vitro screening is often carried out using antibodies that screen for targets based on their structures and not their functions [4].

Various screening models including microorganisms, living cells, tissues, and animals have emerged as alternatives to address the shortcomings of in vitro biosensors [5]. These platforms allow for the monitoring of functions and interactions of target molecules in native environments where diverse biological molecules co-exist, including biomolecules of similar functions. Particularly, living cells offer unique advantages as a low-cost and self-sustainable platform because cell-based sensors are fabricated by simple genetic engineering methods and, once constructed, sensor cells can generate daughter cells with identical sensing capabilities. Furthermore, the unique combination of enzymes and highly sensitive physiological receptor mechanisms present in mammalian cells can be exploited in sensor design. Moreover, many cellular processes can be easily accessed and modified in sensor cells. Therefore, the use of human-derived cells in sensor fabrication has garnered increasing attention, as they are capable of providing functional information about target proteins including their effects on various signaling cascades in the human body without incurring ethical issues [6]. Given these advantages, much attention has been paid to the development of functioning biosensors to screen for biomolecules of interest using live cells.

Biosensors include biological recognition elements and physical transducers, which convert biological interactions into measurable signals. Cellular biosensors are often fabricated by exploiting native receptors or enzymes in living cells as bio-recognition components. Particularly, hard-to-handle receptors such as large membrane proteins can be easily adopted as a recognition element in sensor cells, which provide a natural environment to maintain their stability. The biological interactions or the presence of targets detected by the recognition elements are then converted to readable outputs for external reporting [7]. Among various detection methods, optical detection is often chosen because the genetically encoded optical reporters allow for non-invasive and real-time monitoring of cellular events with a superb spatiotemporal resolution [8,9]. Particularly, the recent development of fluorescent protein (FP) variants has enabled dynamic multicolor imaging of biological events in vivo with improved sensitivity [10]. Optical reporting methods started from simple activation/deactivation of optical signals based on the genomic encoding of fluorescence or luminescence probes and expanded to various approaches due to the superior engineering capabilities of optical probe design. These approaches include (1) fluorescence/bioluminescence resonance energy transfer (FRET/BRET), (2) bimolecular fluorescent/luciferase complementation (BiFC/BiLC), and (3) circularly permuted fluorescence protein/luciferase (cpFP/cpLuc) [11,12,13,14]. While useful, the general limitations of optical detections include photobleaching of FPs and the requirement for external sources of energy and substrates for bioluminescence [15].

The design and construction of state-of-the-art biosensors able to report in vivo activity with improved fidelity require serious protein engineering capabilities. Intein-mediated protein splicing (PS) and protein trans-splicing (PTS) reactions have been widely adopted for designing both recognition and reporter elements, thus providing solutions for many technical hurdles [16,17,18,19,20]. Inteins are intervening protein sequences that are excised from a protein precursor during post-translational modification while conjugating two flanking sequences via an amide bond to yield a mature protein [21]. The implementation of intein-mediated reactions allows for the modulation of sensing elements via the formation and breakage of specific amide bonds to create new recognition scaffolds or for the development of novel or improved reporting methods.

In this review, we aim to provide insights into the characteristics of cell-based sensors with a focus on specific examples of bio-recognition elements and their unique features in terms of screening capabilities compared to in vitro sensing platforms. We will then discuss intein-mediated protein engineering technologies that incorporate various inteins, mechanisms of intein-mediated reactions, and strategies to trigger intein-mediated reactions with external stimuli. We finally describe the use of this technology for designing de novo sensing scaffolds and novel or improved reporting strategies to address the limitations of current sensing approaches.

## 2. Bio-Recognition Elements and Monitoring of Cellular Signaling

Mammalian cells are intrinsically capable of sensing and responding to extracellular signals through native receptors and enzymes involved in different signaling pathways. The development of synthetic biology and protein engineering has enabled the design of custom sensor modules exploiting these proteins. Engineered sensor cells are fabricated by genetic integration of bio-recognition and reporter elements. Representative sensor cells are fabricated using membrane receptors, intracellular receptors, and soluble enzymes, and used for the characterization of enzyme kinetics, cellular signaling, and drug screening [2]. Here, we described representative groups of cellular proteins that are often utilized to build cell-based sensors and discuss their unique characteristics in comparison to in vitro sensing platforms.

### 2.1. Membrane Receptors

Membrane proteins are the first responder to outer cell stimuli and play a critical role in cellular functions by mediating various signal transduction pathways and molecule transport across membranes [22]. Significant efforts have been made to develop biosensors using membrane receptors, as they are important targets for cell biology and biochemical studies, as well as drug screening [23,24]. However, the use of membrane proteins for biosensor fabrication has been limited due to the difficulties associated with handling insoluble proteins and maintaining functional structures in vitro. In contrast, the intrinsic lipid bilayers in living cells provide natural support for membrane receptors, thereby maintaining their 3D structure and their native functions [25,26,27,28]. Therefore, many cell-based biosensors exploiting membrane proteins as a recognition element have been developed to study their functions and behaviors, as well as to screen for target ligands.

**Figure 1:**
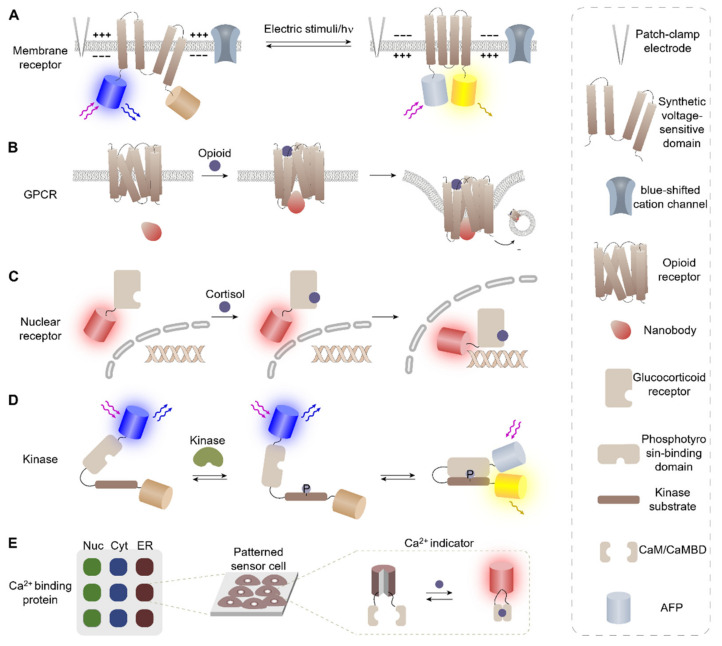
Cell-based sensors developed using various recognition elements. (**A**) The chimeric voltage-sensitive domain is used for detecting membrane potential induced by a patch-clamp electrode or light-gated ion channel. (**B**) Fluorescence protein-tagged nanobodies are used to detect conformational changes in OR and their intracellular trafficking. (**C**) Nuclear receptor GR was used to screen for agonists and antagonists from mixed reagents. (**D**) Kinase activity was monitored using a kinase substrate and its complemented binding domain. (**E**) Ca^2+^ binding domains were used to fabricate genetically encoded Ca^2+^ indicators and Ca^2+^ detecting sensor cells targeting different organelles were fabricated in situ for multiplexed sensing in subcellular compartments.

The activation of membrane proteins is often associated with conformational changes, which can then induce cellular signaling via the gathering of intracellular proteins, kinase activation, and/or transport of signaling molecules. Therefore, current experimental approaches to detect membrane protein activation are often based on detecting conformational changes or translocation of proteins [22]. For example, Monakhov et al. constructed a cell-based sensor that can detect membrane polarization using a synthetic voltage-sensitive domain derived from a voltage-sensitive phosphatase (VSP) isolated from the tunicate *Ciona intestinalis* (Figure 1A) [29]. The sensor protein was designed by introducing a FRET donor and acceptor at each terminus of a synthetic voltage gate protein, in which conformational changes of the protein in the polarized membrane bring the N- and C-terminus of the protein into proximity to activate the FRET signal. The developed sensor cells were first used to monitor membrane polarization induced artificially by direct electric stimuli using a patch-clamp electrode. Afterward, the working mechanisms of natural VSP were studied using sensor cells by monitoring FRET signals upon blue light stimulation. The results of this study indicated that membrane polarization is indirectly induced through Na^+^ transport across a blue-shifted cation channel, CheRiff, upon blue light stimuli. The monitoring of the response cascades initiated by optical stimuli offers a unique opportunity for all-optical electrophysiology, which cannot be easily matched by in vitro methods.

Additionally, Stoeber et al. designed sensor cells that can screen for specific ligands specific to cell surface receptors using an opioid-binding opioid receptor (OR). OR, a type of G-protein coupled membrane receptor (GPCR), was used as a recognition element to screen for the ligand-specific response of receptors. Sensor proteins were designed by taking advantage of the ligand-induced conformational change of OR [30]. The conformational change was reported by fluorophore-conjugated nanobodies, which specifically recognize the ligand-bound form of OR, whose accumulation near the plasma membrane (PM) was observed via total internal reflection fluorescence spectroscopy (Figure 1B). By investigating the ligand-specific activation process of OR, the authors were able to distinguish two different OR activation mechanisms. The OR activation study using sensor cells revealed that peptide and non-peptide ligands function differently. Peptide ligands induced regular activation, including primary PM activation, internalization, and secondary endosomal activation, whereas clinically relevant alkaloid agonists, namely morphine, uniquely drove the third wave of Golgi-localized OR activation in the soma and dendrites.

These examples demonstrate that cell-based sensors are excellent platforms for studying membrane receptors by presenting them in well-folded structures while avoiding the difficulties associated with the handling of insoluble membrane proteins. These studies also demonstrate the applicability of sensor cells for the monitoring of intracellular trafficking by pinpointing the location of specific biological activities depending on the types of ligands.

### 2.2. Nuclear Receptors

Nuclear receptors (NRs) are a large superfamily of proteins that function as transcriptional regulators to control the expression of genes involved in development, homeostasis, and metabolism [31]. Importantly, NRs play key roles in maintaining homeostasis and immune responses and have therefore become important drug targets [32,33]. NRs generally respond to lipophilic substances or steroidal hormones and are activated by gathering cofactors and/or forming dimers, then travel to the nucleus to control gene expression via direct binding to DNA. While various in vitro assays using purified proteins are widely utilized to screen for NR effectors, these assays often fail to provide proper information as the interaction between targets and purified receptors may not be identical to those in biological fluids [34,35]. For example, as in vitro assays lack cofactors and 3D cellular structures, they are not suitable for monitoring of nuclear translocation of NRs.

Several cell-based assays have been developed using nuclear receptors as a recognition element, namely glucocorticoid receptor (GR) [32,33,36]. GR is a constitutively expressed transcription factor that controls many distinct gene networks in response to cortisol or other glucocorticoids. Ligand-bound GRs translocate to the nucleus, where they associate with specific genomic glucocorticoid response elements and assemble transcription regulatory complexes, which activate or repress the transcription of glucocorticoid-responsive genes [34].

For example, Agler et al. constructed a YFP-GR fusion protein to monitor the nuclear translocation of GR when GR is stimulated with agonists or antagonists [37]. The fusion protein was fully localized to the cytoplasm initially and translocated to the nucleus in a ligand-dependent manner (Figure 1C). The sensor cells were used for differentiating a group of steroids including GR agonists, antagonists, and other non-active compounds. The developed sensor rendered information that was otherwise not provided by FP and reporter gene assays, (e.g., the subcellular localization where protein–protein interactions take place), thus providing a cost-effective means for GR effector screening.

Similarly, Ryu et al. fabricated cell-based sensors, which can screen for GR effectors based on fluorescence translocation [38]. GR-fused proteins can serve as reporters of nuclear translocation of GR via signal peptide reconstitution and consequent fluorescence translocation. The developed sensor cells exhibited an outstanding performance in distinguishing between functional and structural analogs, which was difficult to achieve using in vitro sensors. The sensor cells were then used to screen for unknown GR effectors present in natural products such as essential oils and medicinal herbs. Novel GR effectors were identified and their functions in GR-mediated gene regulation were also elucidated, thus demonstrating the capability of sensor cells for screening for biologically active molecules in biologically relevant environments.

### 2.3. Kinase Activity Assays

Kinase signaling networks stringently regulate nearly every aspect of complex cellular processes such as proliferation, motility, and cell survival. Dysregulation of kinase signaling is often associated with various pathologies and, therefore, a comprehensive characterization of the kinase signaling process is essential to understand disease pathologies, as well as for the development of novel therapies. Studying kinase signaling pathways in vitro is rather challenging, as these pathways are highly complex and require various protein–protein interactions, including many receptors and other kinases. However, cell-based sensing technology can successfully overcome these limitations by enabling the real-time monitoring of kinase activity with proper spatiotemporal resolution [39,40,41].

For example, Mehta et al. developed multiple sensor cells that can monitor the activity of kinases by exploiting substrates for various kinases. The activity of target kinases was investigated by detecting the specific binding between phosphorylated kinase substrates and their complementary binding domains (Figure 1D). The phosphorylation-induced protein–protein interaction was reported in real-time via fluorescence activation of cyclic permuted FPs (cpFPs) or FRET, in which the optical signals were activated by the induced proximity between two peptides. Sensor cells were used for visualization of biological activity in real-time while preserving the function and activity of other native kinases. These sensor cells were further engineered to report the activities of multiple kinases simultaneously, thus enabling the monitoring of complex signaling networks via highly multiplexed activity imaging in living cells [41]. The developed sensor cell technology was then applied to an in vivo mouse model, enabling robust tracking of kinase activity dynamics in the cortex of awake mice and revealing a rapid recruitment of highly dynamic neuronal protein kinase A (PKA) activity upon forced locomotion [42].

The protein kinase sensor cells were also widely utilized for the screening of drug candidates [43,44,45,46]. Aberrant kinase signaling and excessive phosphorylation are often reported in tumorigenesis [47]. Thus, kinase inhibitors are considered promising anti-cancer drug candidates and there is high demand for screening platforms that can identify kinase inhibitors for the development of anti-cancer drugs [46,48]. Allen et al. developed a cell-based high-throughput screening (HTS) system to identify PKA agonists and antagonists by utilizing A-kinase activity reporter (AKAR) as a recognition element [44]. The AKAR successfully identified all known agonists among the 160 drug candidates, thus demonstrating its outstanding screening performance. Therefore, HTS assays based on dynamic live-cell activity measurements provide a promising means for high-capacity mechanistic studies and multifaceted drug discovery processes targeting protein kinases.

### 2.4. Genetically Encoded Ca^2+^ Indicators

Calcium ion (Ca^2+^) is a universal second messenger that plays a pivotal role in cell signaling, including neurotransmission. The fluctuation of intracellular Ca^2+^ level exhibits different patterns at different subcellular compartments, generating signals to induce multiple cellular responses [49]. Various types of indicators are available for Ca^2+^ imaging including chemically synthesized and genetically encoded Ca^2+^ indicators (GECIs). GECIs are often built using calcium-binding proteins (CaBPs) as recognition elements in fusion with various FPs or luciferases as reporter elements [50,51,52,53]. Given that GECIs are encoded by DNA, the sensor proteins can be noninvasively expressed in intact tissue including the brain, in addition to targeting specific cell types and subcellular compartments for long-term and repeated monitoring of Ca^2+^ dynamics in vivo [10,54].

**Figure 2:**
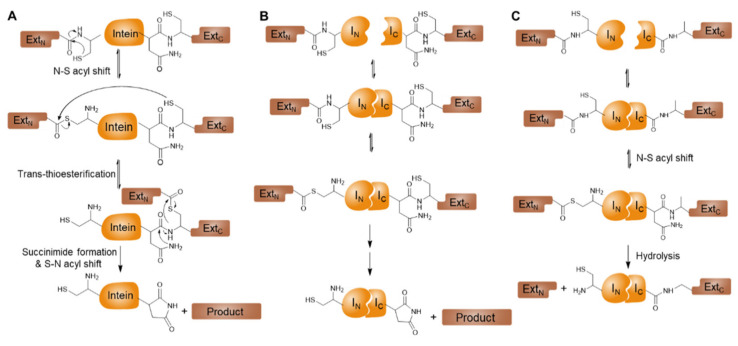
Curved arrow mechanisms of intein-mediated reactions. (**A**) Intein-mediated protein splicing. (**B**) Split-intein-mediated protein trans-splicing. (**C**) Split-intein-mediated protein trans-cleavage. Reproduced with permission from *BioChip J.* **2016**, *10*, 277–287 [58].

Among the various types of CaBPs, calmodulin (CaM) and M13 calmodulin-binding domain (CaMBD) are the most popular and are widely used in conjunction with FRET- and cpFP-based reporting systems to build Ca^2+^ detecting systems such as GCaMP, Cameleon, Pericam, and GECI (Figure 2C). In these approaches, CaMBD and CaM individually conjugated to two FRET pairs or each terminus of cpFP are brought into proximity via heterodimerization upon Ca^2+^ binding to activate the FRET/fluorescence signal. The constructed GECIs have been used for Ca^2+^ imaging in a wide spectrum of organelles, such as mitochondria [55], the nucleus, and the endoplasmic reticulum [56]. Recently, Werley et al. fabricated an array of Ca^2+^ detecting sensor cells that can separately monitor Ca^2+^ influx in ER, cytosol, and mitochondria by patterning lentiviruses, each encoding a different fluorescent reporter conjugated to a signal peptide to localize them into subcellular compartments (Figure 1E) [57]. The array of sensor cells was then used to monitor the correlation between the changes in various physiological parameters including pH, Cl^-^, and NADH in relation to cytosolic, mitochondrial, and ER calcium ion levels. This study enabled the characterization of coordinated calcium signaling in the mitochondria, cytosol, and ER in response to the removal of chemical energy sources for the first time.

Another large advantage of GECIs, particularly GCaMP, is the possibility of cell type-specific expression by coupling to an activatable promotor. For instance, Aryal et al. fabricated sensor cells that can monitor Ca^2+^ signaling in brain astrocytes using astrocyte-specific adeno-associated virus (AAV) in a mouse model [59]. GCaMP variants were used to monitor Ca^2+^ signaling in astrocytes upon cocaine self-administration and exhibited a decrease in the amplitude and duration of astrocytic Ca^2+^ transients in the nucleus accumbens (NAc) shell. Furthermore, the dynamics measured by GCaMP variants localized at PM or ER, respectively, showed that Ca^2+^ activity in the ER was less frequent and shorter in duration than that at the PM, suggesting that near-ER activity is distinct from that occurring at the plasma membrane. As demonstrated by many examples, both cell-specific and sub-organelle-specific Ca^2+^ sensors enable the in-depth characterization of Ca^2+^-mediated cell signaling in vivo.

## 3. Inteins and Intein-Mediated Protein Engineering

### 3.1. Inteins and Intein-Mediated Protein Splicing

Inteins are intervening protein sequences that are excised from a protein precursor during post-translational modification [60]. Upon excision of the intein sequence, the flanking sequences, (i.e., exteins) are ligated via an amide bond and form a mature polypeptide chain. This intein-mediated autocatalytic intramolecular reaction is termed protein splicing (Figure 2A). The canonical mechanism of intein-mediated protein splicing reactions follows a sequence of acyl-transfer reactions, resulting in the cleavage of two peptide bonds linking two exteins to an intein and the formation of a new peptide bond between the N-extein and C-extein. A generalized mechanism of protein splicing involves the following four steps: (1) N to S/O acyl shift, (2) trans-(thio)-esterification between exteins, (3) succinimide formation, and (4) spontaneous hydrolysis of the amino-succinamide residue and S/O to N acyl shift [61].

The first protein splicing domain or intein was discovered in the vacuolar proton-translocating ATPase gene of *Saccharomyces cerevisiae* (Sce VMA) in 1990 [62]. Since its discovery, protein splicing has been harnessed for the development of several protein engineering methods, thus bridging the fields of chemistry and biology and allowing for the otherwise impossible manipulation of protein covalent structures by enabling selective cleavage and formation of amide bonds. The PS reaction is fully spontaneous and does not require any external source of energy or cofactors regardless of the sequence of flanking polypeptides [63,64]. In early applications, the intein-mediated PS was popularly used for expressed protein ligation to introduce synthetic probes to the expressed proteins, as well as for purification of proteins using inteins as cleavable tags. With the discovery of various inteins and expansion of engineering capabilities, intein-mediated reactions became widely incorporated in biosensors fabrication along with other applications. Particularly, the discovery of split-inteins expanded the engineering capabilities by allowing modular approaches to protein modification (Table 1).

The vast majority of inteins function in cis-splicing mode, where the inteins either exist as a single self-splicing unit such as the Gyrase A mini-intein [74] or are linked together with a homing endonuclease such as the VMA intein [62]. A few rare inteins exist in split-form, namely N- and C-fragments of inteins termed N-intein (I_N_) and C-intein (I_C_), respectively. Split-inteins are capable of performing protein splicing in trans when reconstituted and folded together into their active form (Figure 2B) [75,76]. As opposed to naturally split-intein, artificially split-inteins are also obtained by removing the endonuclease domain from cis-splicing inteins and separating the intein halves at the endonuclease insertion positions [77]. Furthermore, some of the naturally split-inteins were further engineered to make smaller fragments, which can be easily prepared by solid-phase peptide synthesis (SPPS) for the incorporation of synthetic probes [71].

A remarkable feature of the naturally split inteins is the intrinsic nature of the two separate precursor fragments to spontaneously self-associate and fold into their active protein splicing state in a complex biological mixture. Particularly, the naturally split intein of *Nostoc punctiforme* (Npu DnaE) is a highly efficient intein exhibiting an extraordinarily high rate of protein trans-splicing compared to other artificial or naturally split inteins [78,79]. Alternatively, the artificially split inteins with reduced activities are often used for the monitoring of protein–protein interactions of sensor domains. The interaction between sensor domains brings fused intein fragments into close proximity to activate the PTS reaction and generate functioning reporter modules such as signal peptides, enzymes, luciferase, or FPs [80]. Moreover, many of these split inteins are orthogonal to each other, (i.e., do not show cross-reactivity with non-partners), thus allowing the combined use of multiple inteins in a single sensor cell [81].

### 3.2. Conditional Protein Splicing (CPS)

**Figure 3:**
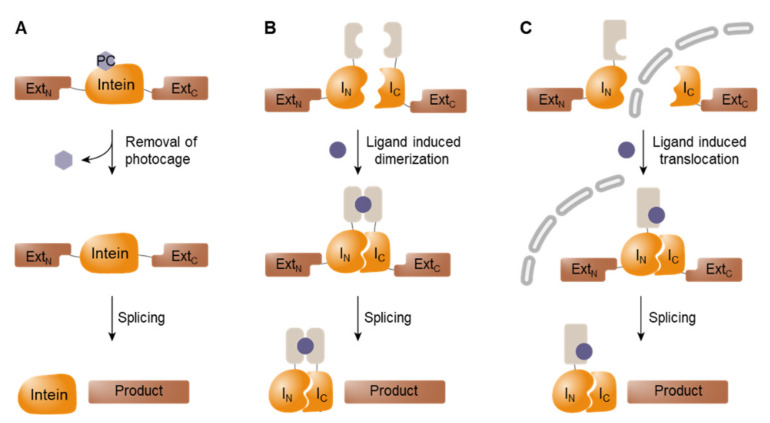
Conditional protein splicing. (**A**) The photocage can be removed by light stimuli to activate intein-mediated reaction. (**B**) Low-affinity split intein pairs are activated by induced dimerization. (**C**) Spatially separated split inteins are activated by translocating molecules into the same cellular compartment.

Split-intein-mediated PTS systems offer a versatile tool for the fabrication of cell-based sensors [63]. Particularly, conditional protein splicing (CPS) systems in which the PTS reaction is activated via user-specific triggers have been widely utilized for the design of bio-reporter modules [82]. Figure 3 illustrates the different types of CPS systems available. First, external stimuli can be used to activate initially inactive forms of inteins. One method utilized for inactivation is the introduction of a photocage on the penultimate residue via SPPS or incorporation of non-canonical amino acids by using an orthogonal tRNA and tRNA synthetase, (i.e., amber codon suppression technology) (Figure 3A) [83]. In both systems, the light-triggered removal of photocages restores the activity of split inteins, thus enabling the activation of proteins in a spatially targeted manner.

Additionally, low-affinity split inteins can be activated by the induced proximity of two fragments, which is often accomplished by fusing two binding pairs to each split intein (Figure 3B). For example, VMA inteins fused to the hetero-dimerization domains of FKBP and FRB mediated PTS in the presence of rapamycin, which stimulates the dimerization of interacting domains and thus brings intein fragments into close proximity [84].

Third, co-localization of the initially separated intein fragments also triggers splicing. When two intein fragments are spatially separated in different cellular compartments, the binding of split fragments is physically banned to suppress the splicing activity (Figure 3C). PTS can be activated by triggering the translocation of a split fragment to co-localize both intein fragments into the same compartment, in which they react spontaneously [85]. This approach can be further engineered to shuttle in an inactive form of intein to a targeted destination where the inteins are activated upon arrival, thereby mitigating off-target activity [85].

## 4. Application of Intein-Mediated Reactions in Cell-Based Sensor Fabrication

Intein-mediated protein engineering, especially split-intein-mediated PTS, offers new opportunities for the fabrication of cell-based sensors. There are several unique advantages of intein-mediated reactions, making them a useful tool in sensor cell preparation. First, inteins can be introduced to sensing units through fusion proteins via genetic modification. They can also be easily incorporated into existing sensors to enhance their performance without requiring complicated re-design steps. Moreover, inteins mediate highly specific splicing reactions in physiological conditions without a need for an external source of energy or cofactors, thus minimizing disturbances on sensor cells. Further, intein-mediated protein engineering allows for modular approaches to enable the easy exchanges of sensing and reporting elements, thus widening their applicability. Finally, intein-mediated PS and PTS both generate seamless target proteins conjugated via a stable amide bond to support the fabrication of biosensors with high target sensitivity and bio-stability. Due to these advantages, inteins have been widely exploited in sensor fabrication to generate sensors with unique capability and/or improved performances.

### 4.1. FRET-Based Detection System

Single-molecule Förster resonance energy transfer (FRET) from the excited-state donor fluorophore to the acceptor fluorophore occurs when two fluorophores with overlapping emission/absorption spectra are located in close proximity [86]. As FRET is sensitive to the distance and orientation change between two fluorophores on a nanometer scale, it is well suited for studying biomolecular events such as protein folding and protein–protein interactions by extending the capability of fluorescence microscopy [87]. Particularly, FRET constitutes an unmatched tool for the fast monitoring of molecular processes at a millisecond scale [88]. Thus, FRET-based reporters are often utilized for monitoring protein–protein interactions in cell-based sensing by enabling quantitative analysis via ratiometric imaging [89].

**Figure 4:**
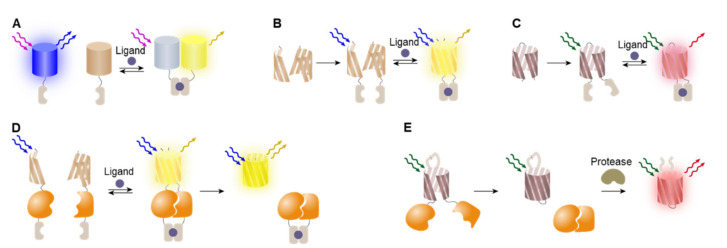
Reporting elements to fabricate cell-based sensors. (**A**) Cell-based sensor design based on FRET. (**B**) Cell-based sensor design based on BiFC. (**C**) Cell-based sensor design based on cpFP. (**D**) Intein application for BiFC with reconstitution of split FP. (**E**) The intein is applied to enhance FP formation.

FRET-based approaches are often used to design sensor cells in which the targeted interactions induce changes in the conformation or assembly status of sensing elements (Figure 4A). Broad categories of cellular events are associated with conformational changes, such as binding of ligands and post-translational modifications, (e.g., methylation or phosphorylation). For example, Miyawaki et al. constructed a calcium ion (Ca^2+^) indicator based on intramolecular FRET, in which sensor cells were genetically encoded to express tandem fusions of donor AFP, calmodulin, M13 CaMBD, and acceptor AFP. The binding of Ca^2+^ makes calmodulin wrap around the M13 CaMBD to bring two FRET pairs into close proximity to have efficient energy transfer [90]. Similarly, Kunkel et al. studied serine/threonine kinase protein kinase B (PKB), which is a critical regulator of insulin signaling, cell survival, and oncogenesis [91]. To study PKB signaling in live cells, they generate cell-based sensors using FRET called B kinase activity reporter (BKAR). BKAR consists of donor and acceptor fluorophores conjugated to each end of the sensing domain, in which the FHA2 phosphothreonine binding domain and a consensus PKB phosphorylation sequence (RKRDRLGTLGI) are conjugated with a proper linker. Phosphorylation of the tyrosine in the substrate sequence induces conformational changes via binding between the FHA2 and PKB phosphorylation sequence to activate the FRET signal. The BKAR was used to identify specific kinase-substrate pairs and to monitor PKB signaling in the cytosol and nucleus, revealing that the t_1/2_ of the BKAR maximal response in the cytosol was approximately three times faster than that of the BKAR response in the nucleus. These findings indicated that the delayed PKB signaling in the nucleus likely reflected the time it takes for activated PKB to translocate into the nuclear compartment.

While useful, there are some limitations associated with FRET-based detection system including low FRET yield, photobleaching, autofluorescence, phototoxicity, and undesirable stimulation of photobiological processes [82,92]. Arguably, the largest limitation of FRET measurements is the low signal-to-noise ratio (SNR) associated with FRET imaging. While many technologies have been developed to measure FRET, all of them generally suffer from poor SNR when compared to imaging of a single fluorescent label [82]. Many reports have discussed various approaches to overcome these limitations. For example, bioluminescence resonance energy transfer (BRET), in which the donor is substituted with luciferase and the acceptor fluorescent protein is excited by luciferase luminescence to show an emission, was developed to avoid the complication associated with the choice of excitation light [89]. Intein-mediated approaches were also tested to overcome these limitations.

For example, Borra et al. utilized FRET quenched split inteins to monitor the progress of transcription factor YY1 protein labeling via a semi-synthetic approach [93]. In their approach, I_C_ was synthetically prepared to carry both a quencher and a fluorophore in the same molecule. When the FRET-quenched I_C_ reacted with a fusion of YY1-I_N_, the quencher was separated from the fluorophore, which was covalently conjugated to YY1. This is a background-free labeling approach based on FRET quenched fluorophore and can be utilized for protein modification to control their cellular localization and potentially alter their biological activity. Similarly, Lee et al. generated a stable, matrix-sticky, and protease-sensitive extracellular reporter for the monitoring of matric metallo-protease 2 (MMP2) activity in live cell culture [94]. Their sensor comprises a collagen-binding protein and a FRET pair conjugated via an MMP2-specific substrate. The effective FRET pair was constructed by introducing a synthetic fluorophore to AFP via split-intein-mediated reaction. Using this reporter, the activity of mutant and wild-type proMMP2 was investigated, suggesting that this approach could potentially be used for the analysis of protease-related extracellular signaling and tissue remodeling.

### 4.2. BiFC-Based Detection System

FPs have a β-barrel structure consisting of 11 antiparallel β-strands and 10 α-helix loops surrounding a chromophore assembled during the post-translational process [95]. As the chromophore is protected by the β-barrel structure, disruption of the β-barrel structure via protein fragmentation results in the loss of fluorescence activity. By splitting the AFP at the seventh loop located between the seventh and eighth strands, Hu et al. were able to design two split fragments of AFPs without fluorescence activity, which possess the intrinsic ability to properly refold into a beta-barrel structure when brought into proximity, thus restoring the fluorescence activity [96]. This approach, named bimolecular fluorescence complementation (BiFC), is often adopted for designing cell-based sensors for screening protein–protein interactions. Briefly, two proteins of interest (POIs) are introduced to each fragment of AFPs, respectively, and the complementation of split-AFPs co-expressed in a sensor cell can be induced by the interaction between the two POIs (Figure 4B) [97]. Since the establishment of this basic concept, many efforts have been made to improve the performance of BiFC by utilizing the expanded color palettes of AFPs, as well as luciferases. Both BiFC and BiLC offer useful strategies for screening protein–protein interactions. However, this approach has some limitations that must be addressed, including high background noise and false positives originating from spontaneous assembly [13,14].

The intein-mediated PTS reaction offers an improved approach that enables the reconstitution of split-reporter molecules instead of reversible complementation and can be used for screening protein–protein interactions (Figure 4D) [8,98]. In this approach, intein fragments are inserted between the recognition elements and BiFC reporters to induce protein trans-splicing upon the delivery of stimuli, resulting in covalently conjugated full-length reporters [9]. The reconstituted seamless reporter proteins show significantly enhanced signal intensities with low background signals compared with complemented split-reporters. Additionally, intein-based sensors can easily be modified due to their modular design, allowing the easy exchange of sensing and reporter domains [98].

Similarly, Kim et al. used this approach to monitor the activity of nuclear receptors using the Ssp DnaE intein in combination with a split Renilla luciferase (RLuc) and a glucocorticoid receptor (GR) [19]. The sensor was fabricated by fusing GR with the C-terminal halves of RLuc and DnaE, which localizes in the cytosol. A second fusion protein containing the N-terminal halves of RLuc and DnaE was localized in the nucleus by adding a nuclear localization signal. Once corticosterone induces GR translocation into the nucleus, the C-terminal RLuc interacts with the N-terminal fusion protein located in the nucleus, thereby reconstituting the full-length RLuc by spontaneous PTS. These sensor cells provide a quantitative bioluminescence activity that can be used to determine the amount of GR translocated into the nucleus, and have been used to detect corticosterone levels in mice.

### 4.3. Circularly Permuted Proteins for Biosensors

The circular permutation of fluorescence proteins provides an additional fluorescence-based reporter system for cell-based sensors. cpFPs are generated by linking the original N- and C- termini with a flexible linker and introducing new N- and C- termini either in the midpoint of the β strand 7 or in surface-exposed loop regions [99,100]. Circular permutation breaks the β-barrel structure, resulting in fluorescence turn-off, which can be turned on by bringing the new N- and C-termini into close proximity (Figure 4C) [101]. Reporter units based on cpFP are often utilized in the fabrication of cell-based sensors by introducing two POIs to each newly introduced termini of cpFP to monitor protein–protein interactions. This approach has several advantages, as cpFP-based probes have lower molecular weight, which facilitates the optimization of expression rates and subcellular targeting, and do not suffer from the variations in pH sensitivities or maturation rates of two FPs compared to FRET-indicators [102]. Additionally, the combination of cpFPs of different emission spectra made it possible to perform multiplexed assays in a single cell. Mehta et al. demonstrated that six cpFP probes that were co-expressed in a sensor cell could selectively respond to sequential stimulation with Fsk/IBMX, EGF, and histamine. These single-fluorophore biosensors represent a powerful platform for dissecting complex signaling networks via highly multiplexed activity imaging in living cells by enabling the simultaneous monitoring of several biochemical events [41]. Multiplexing is critical for understanding the interplay between fast processes when combining data from independent experiments becomes challenging.

While useful, the aforementioned approaches have several limitations that must be overcome: (1) difficulties associated with designing functioning probes with sufficient brightness; (2) the variation in emission effectiveness of cpFPs depending on the fusing partners and linker composition, and (3) the long maturation time required after complementation [102]. Intein-mediated approaches address these issues by providing a reversed approach for the implementation of the cpFP platform. Guerreiro et al. developed a genetically encoded switch-on fluorescent biosensor consisting of a cyclized GFP, termed cVisensor, with an adenoviral protease cleavable site as a switch [103]. In this approach, cyclization was carried out by fusion of I_C_ and I_N_ at the N- and C-terminus of a GFP harboring a protease cleavage site to prepare a nonfluorescent cyclic mutant (Figure 4E). cVisensor was then utilized for live cell monitoring of adenovirus infection, as the intracellular biosensor is specifically activated by the viral protease. This new scaffold based on cyclization and cpFP showed a promising potential for basic research in virology and biotechnological applications of recombinant virus biopharmaceuticals.

### 4.4. Cyclic Peptides as Biosensing Scaffolds

Cyclic peptides are a very unusual class of biomolecules with a robust backbone and are increasingly utilized in various applications including drug discovery and biosensing. The split-intein-mediated circular ligation of peptides and proteins (SICLOPPS) is a genetically encoded method for the intracellular production of cyclic peptides [104]. Cyclic peptides can be easily prepared in live cells by expressing I_N_-target protein-I_C_, in which I_N_ and I_C_ come together to form an active intein that splices to give a head−tail cyclized peptide, making an attractive scaffold for sensor fabrication [61,105]. The circularized peptide often loses its natural activity due to structural restriction, and the activity could be restored via linearization of the circular peptide. This system is often interfaced with optical reporters and used as a trigger to activate an optical signal. Sakamoto et al. designed a novel BiFC system using self-assembling split-GFP, in which a C-terminal fragment was covalently cyclized via a caspase-3 substrate sequence (DEVD) mediated by split intein [106]. Sensor cells monitoring caspase-3 activity were fabricated using this system, as the specific cleavage of the cyclic C-terminal fragment by caspase-3 induces the GFP reassembly and fluorescence recovery. This novel reporter showed a 100-fold increase in signal-to-noise ratio compared to current BiFC methods. This enhancement was possible because the cyclic GFP fragment prevents false-positive signals from unexpected self-association between two split GFP fragments.

Similarly, Kanno et al. developed backbone cyclized luciferase to monitor protease activity utilizing split-intein-mediated PTS [107]. The biosensor was based on a backbone cyclized luciferase protein reporter containing the caspase-3 recognition sequence DEVD. When cyclized, the luciferase reporter was enzymatically inactive due to the steric strain imposed by the backbone cyclization. In the presence of caspase-3, cleavage of the cyclized peptide restored the activity of the luciferase, allowing real-time detection of caspase in live mice in real-time. Both intein-mediated cyclic protein fabrication approaches enabled the sensitive monitoring of protease activity, demonstrating improved performance in comparison to conventional BiFC or BiLC with minimized background signal.

### 4.5. Intein Zymogens for Protease Activity Monitoring

Recently, caged intein zymogens have been proposed as biosensors for proteolytic activity [80]. Gramespacher et al. first prepared a caged version of Npu DnaE split inteins by converting two fusion proteins of residues 51–102 of I_N_ to full-length I_C_ (Npu_C_Cage) and residues 1–13 of I_C_ to full-length I_N_ (Npu_N_Cage). They proposed that these cage sequences would participate in intra-steric interactions with the split intein fusion partner, effectively locking each half of the split intein in its binding intermediate structure and thus restricting fragment association and splicing. The authors demonstrated that the genetically encoded caged split intein pairs can be activated by targeted proteolysis and creating split intein zymogens, and suggested that this platform can be extended to various orthogonal split intein pairs to create CPS sensors. For example, a specific protease cleavage sequence was introduced between each native split intein and its corresponding cage sequence, and triggered split intein association by proteolysis, resulting in the generation of the desired protein reporter. This system was tested using a TEV-activated split GFP intein zymogen system that was able to produce GFP in the presence of the TEV protease in live cells [80].

### 4.6. Fluorescence Translocation Sensor via Signal Peptide Reconstitution

Intein-mediated reconstitution of signal peptides has been uniquely utilized for fluorescence translocation biosensors, in which fluorescence proteins are translocated to a predetermined subcellular site rather than altering the photophysics of FPs [38,85,108,109]. Signal peptides are short peptide sequences with 16–30 amino acids and deliver proteins to target subcellular organelles [110]. The activity of signal peptides is determined by their sequence alone regardless of their tertiary structure, and can therefore be coupled with intein-mediated reaction. The characteristics of intein-mediated reactions are the formation and the cleavage of specific bonds. Unlike proteins, split signal peptides do not bind to each other to generate false-positive signals nor do they require refolding after reconstitution to yield false-negative signals, thus making them an ideal target for intein-mediated activation. Target specific activation of signal peptides has been utilized for building cell-based sensors to monitor cell signaling molecules such as Ca^2+^ and glucocorticoids based on intein-mediated protein splicing, as well as intein-mediated protein cleavage [85].

**Figure 5:**
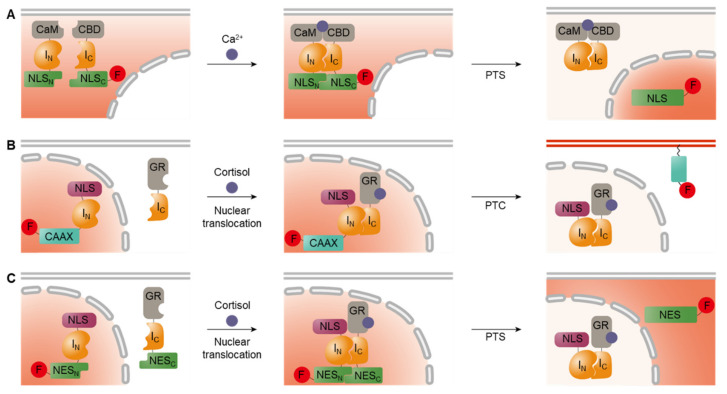
Cell-based sensor design via signal peptide reconstitution. (**A**) Nuclear location signal (NLS) peptide reconstitution triggered by CPS. (**B**) Activation of CAAX tag induced by CPC. (**C**) Nuclear export signal (NES) peptide reconstitution triggered by CPS.

Jeon et al. fabricated both Ca^2+^ and glucocorticoid detecting sensor cells by using split-intein-mediated protein splicing and protein cleavage reactions, respectively. The key strategy of this method is the activation of signal peptides via the formation of the cleavage of the amide bond. The activated signal peptides induce fluorescence-translocation with the target molecule as an activation indicator. First, the Ca^2+^ detecting sensor cells were built by conjugating CaM and CaMBD to each split-VMA intein and attaching split-fragments of the nuclear localization signal (NLS) to the other end of the inteins. An FP was also introduced on one end of the fusion protein as a reporter. Then, the Ca^2+^-induced heterodimerization of these two proteins triggers CPS, resulting in the reconstitution of the NLS peptide, which translocated the fluorescent signal to the nucleus to report the presence of the target. This system was then used to monitor histamine-induced Ca^2+^ signaling, indicating that the sensor cells are also capable of detecting induced Ca^2+^ signaling (Figure 5A) [85]. Alternatively, glucocorticoid detecting sensor cells are designed by using glucocorticoid receptor (GR) as a recognition element and are based on the intein-mediated protein cleavage reaction. For sensor fabrication, an inactive C1A mutant of C-extein was used to prompt protein cleavage instead of protein splicing (Figure 2C). Fusion proteins of GR with Npu-I_C_ and Npu-I_N_ fused to mCherry and CAAX tags on its N-terminus and an NLS sequence on its C-terminus were prepared and initially located in the cytosol and nucleus, respectively, where the CAAX tag is a PM-targeting sequence and is activated when exposed at the C-terminus. The activity of spontaneously reacting Npu intein was prevented by locating fusion proteins in different cellular compartments, after which the intein-mediated reaction was triggered by the presence of glucocorticoid, which binds to GR and initiates nuclear transport (Figure 5B) [85]. This sensor was used to identify GR effectors from structural analogs without GR binding activity. This sensor was further engineered as a rapid response sensor, which can report the presence of targets within 5 min, by exploiting the split nuclear export sequence (NES) peptide and the developed sensor cells were utilized to screen for GR effectors in natural products including essential oils and medicinal herb extracts (Figure 5C) [38]. Through screening experiments, the authors were able to identify novel GR effectors from essential oils and medicinal herbs, indicating that the cell-based sensors could lay an excellent foundation for drug screening [38,109].

## 5. Conclusions and Perspectives

This review discussed various approaches to making biosensing platforms out of living cells, which are capable of screening for targeting biological events in their native context with improved sensitivity and specificity. Cell-based sensors provide a robust and self-sustainable platform, which provides a powerful tool for various types of screening, including biomarker detection, studying enzyme activities, and cellular signal mapping. Particularly, sensor cells based on genetically encoded Ca^2+^ indicators were prepared and used for visualizing cell type and organelle-specific Ca^2+^ signaling in vivo. Many types of cellular proteins were also adopted for the fabrication of sensor cells including membrane receptors, nuclear receptors, and soluble kinases, enabling the monitoring of site-specific activation of receptors and kinase signaling cascades. These sensor cells all utilize optical reporting systems based on AFPs and luciferases. Although many excellent reporting units are available, there are also some limitations that must be addressed, including low fluorescence efficiencies, high background noise, and the fragile nature of the reporter. To address these issues, many efforts are being made to design novel or improved sensor modules with enhanced capabilities. Intein-mediated protein engineering offers a robust tool for protein engineering in vivo by allowing the formation and breakage of specific peptide bonds without the requirement for external sources of energy or cofactors. Intein-mediated strategies are suitable for biosensing applications because inteins can easily be incorporated into sensing modules via simple genetic modification. Additionally, intein-mediated reactions are fast and deliver high yields regardless of flanking sequences. Furthermore, inteins allow for a modular approach, thus facilitating the replacement and exchange of sensing modules. This review provided many examples of intein-mediated reactions used for the generation of new sensing scaffolds and the improvement of current reporters. These approaches have enabled the generation of more robust and versatile sensing platforms, which can expand the applicability of cell-based biosensors for real-time in vivo monitoring of biological events of interest. The future outlook of cell-based biosensors for medical applications is particularly prominent as the human cell-based sensors could be generated in situ in the human body or be implanted in the donor for personalized medicine. Several examples show the future potential of cell-based sensors as a theranostic agent possibly in conjunction with CAR-T cell therapy [111]. The novel theranostic sensor cells would provide next-generation personalized bio-therapeutics with a strong in situ diagnostic capability. The ability to perform continuous, long range monitoring would extend the usefulness of sensor cells as a theranostic platform. Additionally, consideration of controlling the production and secretion of therapeutic materials is another requirement. The advanced engineering capability with an adequate quality control is necessitated to effectively load complex, higher-order functions into sensor cells for successful exploitation of sensor cells in clinical settings.

## Figures and Tables

**Table 1:** Inteins of various characteristics for intein-mediated protein engineering.

Intein	Type	k_splice_ (s^−^^1^)	t_1/2_	Yield (%)	Ref.
*Mxe* GyrA	Contiguous	1.9 × 10^−5^	10 h	>90	[65]
*Pab* PolIII	Contiguous	1.6 × 10^−5^	12 h	74	[66]
*Mtu* SufB	Contiguous				[67]
*Ssp* DnaE	Naturally Split	1.5 × 10^−4^	76 m	<50	[68]
*Npu* DnaE	Naturally Split	3.7 × 10^−2^	19 s	>90	[68]
*Ssp* DnaB	Artificially Split	9.9 × 10^−4^	12 m	32–56	[69]
*Sce* VMA 1	Artificially Split	1.2 × 10^−3^	10 m	61–73	[69]
*Mtu* RecA	Artificially Split	NA	60–120 m		[70]
AceL TerL	Naturally Split	1.7 × 10^−3^	7.2 m	90	[71]
*Ssp* DnaB S1	Artificially Split	4.1 × 10^−5^		40–45	[72]
*Ssp* GyrB S11	Artificially Split	6.9 × 10^−5^		80	[73]

## Data Availability

Not applicable.
